# An Ideal College Curriculum

**Published:** 1891-09

**Authors:** Milton F. Ault

**Affiliations:** Indianapolis


### THE DENTAL REGISTER.

VOL. XLV.] SEPTEMBER, 1891. [No. 9.

### COMMUNICATIONS.

### An Ideal College Curriculum.

BY MILTON F. AULT, M. D., D. D. S.

INDIANAPOLIS, JUNE 30, 1891.

*Mr. President and Members of the Indiana State Dental Association:*

The assembling of members of a profession in local, State or national association ought to indicate a laudable purpose. He who has laid aside his business, feeling that dentistry should have an individuality; that we need more power to follow our convictions; that the empiric and mountebank should be subdued and honor and ability promoted, and that the memory of fraternal intercourse is needed to cheer in seasons of darkness, is the man whose purpose in coming here is that of loyalty and devotion. What a beautiful thing it is to have a proper conception of the duties incident to this life, and then have the wisdom and fearlessness to be loyal. How much the terms devotion, fidelity and love are abused by misapplication. He who has seen his convictions of life outraged, who has felt the impulse which springs from home association, who has experienced the dread and anxiety preparatory to battle, and who has, for the sake of manly independence and pure government, dedicated to his country all he holds sacred, appreciates the power and sublimity of loyalty. He who has striven through poverty and persecution, guided by the right as he understands it; who has been gentle, respectful and unassuming, feeling an intense disgust for bombast, glitter and deception; who has sustained the moral, intellectual and religious man because God expects it, and who has given value received, because conscience exacts it, is the man who feels the power and grandure of loyalty to his employment. While our mission here is to discuss the merits of the many modes practiced, and to consider the feasibility of methods which exist only as theories, we must not overlook the far-reaching subject of the true training of the young men and women in our offices and colleges, who are working for membership in the dental profession. The man who is accustomed to doing a thing in a certain way listens to theory impatiently, but all the good which we enjoy to-day once had the theoretical form. Theory contains revolution —it upsets old practices and drives men from the intrenchments which their cunning and selfishness have devised toward the ideal in civilized life.

When a person enters a community and it becomes known that he professes the knowledge and delicacy of manipulation that belongs to dentistry, the people expect him to extract teeth with superior skill; that after an operation there shall be a reasonable service; that after inserting an artificial denture, that which was lost in expression, shall be restored. Anything short of this is not dentistry, and the college that fails to equip its students in these particulars fails to meet a very just and reasonable expectation from the people.

When one proposes to practice dentistry, it is not only the better dentists in that locality, but well-qualified, sensible dentists everywhere that expect him to deport himself in a professional manner. He is to live above those allurements which, in the end shatter health, render him worse than a beggar, and make his profession infamous in the estimation of refined people. He is not to startle the populace by announcing a revolution in dentistry —wonderful performances—as painless extraction, fifty-cent fillings, three-dollar teeth, work guaranteed, and vitalized air. The profession desires him to cultivate that dexterity and skill that will make his work the only advertisement needed—and learn that milk ticket, grocery sack and scioptic notices are the schemes employed by the ignoramus to defraud the penurious and the unsuspecting poor.

The profession expects him to be contented with the plain and unassuming word dentist, and if he administers to the wants of his patients with the intelligence and sympathy that he should possess, he will find in that word an all-sufficiency. Anything short of this is not decorous, and the college which fails to inform its students in the details of deportment fails to meet a very just and reasonable expectation from the profession.

When the duties incident to dental practice are chosen as a life-work, the man desires to close his life with a considerable degree of financial success, besides throughout his career the divine and human governments under which he lives demand a part of his time and consideration. To succeed financially, is the cardinal aim of most men, and how to achieve this without too much sacrifice on the part of the physical, mental and religious man is a very important question. It is very evident to one of experience that with a low scale of prices the practitioner suffers in every particular. Those who are satisfied with only the best work that art can give, know how exacting our operations are, and with what care and tension of nerve they must be executed, so the attempt to be noted as a conscientious and at the same time cheap operator is sure to end in physical and financial ruin. With the knowledge that we have of superior dental service, we can not work from sunrise to sunset unless it is done at the expense of constitution and mind. Continuous effort, although legitimate in itself, and may seem necessary, means either a short life, misery in old age, an uncultivated mind and hopeless future, because in our struggle to satisfy the money aim of life we take from other faculties that which has been ascribed to them through divine order. This may seem like a wide departure from the subject, but when we consider our obligations as men and the purpose of the creator, we can not escape the conclusion that the religious and political sides of our natures are just as pertinent themes for a dental society as gold crowns and painless operations. I suppose there are but few men who are fully conscious of the different phases of a dentist's life but what have often closed office and trudged home, feeling too tired for exercise, and no disposition for social or intellectual improvement. These hampering and cramping, selfish and forbidding tendencies in our lives it is the business of the college to guard against.

Now, when we consider what the people and the profession expect, what the human and divine governments expect, and what we anticipate for ourselves, there is sufficient ground for demanding a more generous training from those who pose as preceptors and professors, and the student who tries to assume the responsibilities of his own office without a thorough preparation, will soon feel the weight of disappointment, misery and neglect. Any man or woman that possesses the natural ability to become a dentist, has sense enough to desire a reputation for superior work, and honest enough to give value received, so when he faces the stern barriers of actual business and finds that the babbling and many irrelevant things which have been forced upon him as essentials in instruction, and which have cost him many hard-earned dollars, does not enable him to meet the requirements which we have cited, the college which sent him adrift will surely be the object of his invective and the recipient of many harsh but merited accusations. The principle in educational economy, "that whatever is to be learned ought to be learned by doing it," has a particular import in dental college instruction, and no student, no difference what his rank or relations may be, should be graduated until he is able to perform the operations that are incident to dental experience, and conduct his practice in a Christian and ethical manner. Being the son of a dentist should entitle him to but little more consideration than is accorded others, and the M. D. degree is often counted for more than it is worth in determining the pupil's fitness for the special work of dentistry. While I am especially desirous of ornamenting, and fortifying our profession with the intelligence and refinement that pertain to other degrees, the possession of that mental and manual training which is embraced in the degree D. D. S., is the supreme topic for faculties and examiners. When the characteristics of the ideal dentist are considered, we naturally ask what kind of material should be admitted to the college? What should be taught? In what order should it be presented? What method should be employed, and what should be the qualification of him who presumes to teach?

An ideal course of instruction can be attained by assembling those elements which are so free of defect as to answer the divine and human, professional and personal expectations, and such a curriculum followed by a well-balanced student, as presented by a faculty keenly alive to its duty, could not fail to produce the type of character which is so essential in sustaining the respect and influence and power of our profession. Let us now briefly consider the conditions upon which a pupil may be admitted into a dental school. While it is clear that a school of this kind is established for a specific purpose, the conditions governing the admission of students differ but little from those adopted by other schools. Our terms have no respect to locality, age or sex—a condition with which I can readily comply. We claim that a certain degree of general scholarship shall be the principal element of elligibility.

To this condition I heartily agree, but I regret the loose manner in which it is often applied. It may seem to the crude notions of the applicant that the description of the Amazon, the boundary of Massachusetts, the analysis of a sentence, or the spelling and pronunciation of a word has a very obscure relation to preparing a cavity or making a crown, but the teacher who has instructed in the details of this specialty knows full well of the attention, interest, perseverence and aptness of the mind that has the discipline of a general education, and the awkwardness and embarrassment of the pupil without such training. True, a learner with patience and natural ability may acquire the power of building beautiful things in gold without knowing about the physical features of his own country or that he lives in 40° N. latitude, but the mission of the dental school is to replenish our depleted ranks with men—men whose ideals are sufficiently elevated to make them desire to know about the partitioning of Africa, the great political struggles in the world, and the facts of sacred history. This it is that rests and stimulates when we are weary from the exactions of our special employment.

The conditions of admission, so far as I have observed, have no reference to those characteristics of the student which are so essential in making a full grown, manly, professional man. These personal traits may be considered in the order of health, natural ability, love of learning, an elevated ideal, self-reliance, perseverance, patience and humility. These and other qualities are usually mentioned in describing a desirable student in general school work, so I have endeavored to select those terms which I think would have a particular meaning in describing a desirable applicant for admission into this special work. Not long ago a young man came to my office. He said that he wished to study dentistry, and that he would like to stay with me till the beginning of the coming session. He told me what kind of a life he had been living; *i. e.*, as to his occupation, the extent of his general education, etc. But there was one thing he did not mention. I saw in his large glossy eyes, sallow complexion and general carriage, something which caused me to discourage his pursuit. I was very well acquainted with his family history, was well pleased with his explanation, and would have felt proud to have his name on the roll of our college, but I did not feel justified in encouraging him to begin a work which I was certain he was physically unable to do, and which would have hurried him to his grave, a victim of tuberculosis. In this instance I think I did what the college should do with similar cases. Of course it would be difficult for the school authorities to determine with reference to the applicant's character without the aid of a preceptor, and in securing the desired information it would be well for those knowing most about the pupil to answer the following:

Do you believe the young man whom you recommend possesses the natural ability to become a desirable member of the dental profession?

From your observation, does he love learning to the extent that we may expect an earnest effort to obtain knowledge?

Is it your opinion that his ideal is sufficiently elevated to make him appreciate the purpose of his undertaking, and desire the dignity of a cultured character?

Is it his custom to think for himself?

Have you noticed in him the concentration and enthusiasm that enables one to surmount great difficulties?

And does he possess the patience and humility, and realize his own littleness sufficiently to be teachable?

Now, I have tried to set forth briefly the characteristics of the embryo dentist, and to indicate the quality of material that should be placed under the care of the faculty. These conditions may be said to exact too much, but I believe that for a special work we should demand special material, and by so doing the college can offer no excuse for disappointing the profession or the public.

The student that we need is now introduced, and the next important question is, What shall he be taught?

We can answer this best by discussing the nature of dentistry and the expectations which the school is to satisfy. Dentistry is of a triple nature. It is a specialty in medicine, a specialty in art, and a specialty in mechanics. Our students are not to become general practitioners. They are not to become sculptors, neither are they to be mechanics, but they are to treat and operate upon certain organs of the human body which consist of living tissue. Their *materia medica* embraces probably sixty medicinal agents, and their instruments are truly surgical. They are to meet æsthetic requirements by means of the mind and imagination, and in this respect they engage in the refined arts.

Their manual and bodily efforts are dictated by a disciplined mind, and in this their work is largely mechanical. To know the anatomy, physiology, histology and chemistry of the organs upon which they operate; to know the relation of these organs to the rest of the organism; to understand the properties of the material they use; to know what to say and what to do, and how to do, are among the important things for their study. While I am in favor of pupils studying subjects that are remotely related to their work, I am opposed to worrying about the foramina, surfaces and ossification of the temporal bone, if it must be done at the expense of the peridental membrane or development of the teeth. I am not in favor of memorizing the origin, different preparations, and antagonists of digitalis, when it is necessary for the time to be given to anæsthetics. They should not study iron until they are thorough in the manipulation of gold. In short, I believe the mission of the dental college is to make dentists, and that there is no legitimate excuse for the school which sends a pupil forth feeling that he is not competent for the work. Essential knowledge first, and ornamental knowledge afterward, should be the controlling thought. This brings us to the modes of study.

The progress of the student will be determined by the place he begins, the number of things he undertakes in a given time, the order he observes, the way he understands, and the permanency of what enters his mind.

The object of the learner is not to find out something for himself, but rather to acquaint himself with what is already known; so I believe that the place for him to begin is on the first page, the teacher taking but little for granted. The principal difficulty with students generally is that they undertake too much, but it is to be hoped that the three-year course will obviate this difficulty.

Students generally appreciate a logical order in study, and this is proof that their progress will be more marked if it is observed in arranging work. To talk to the pupil concerning Kingsley's case of irregularities would awaken little interest, but to present: 1st, The proper period for regulating; 2d, Impressions and models; 3d, Mechanical forces, etc., could not fail to attract attention. Lastly, if the pupil wishes to understand, he must give proper heed to the language used as well as the thought, and must not be satisfied until he is master of the situation.

Having the pupil with the requisite disposition, having the proper selection of subjects and the learner with the true conception as to the mode of study, is very essential, but can not attain the end for which the school was created, unless the management fully appreciate the meaning of method. A person about to undertake the study of dentistry in looking over the various college announcements would conclude that the true value of a school depends upon age, well-arranged buildings, immensity of museum, completeness of apparatus, number of instructors and size of clinic, but a person after short contact with an institution awakes to the fact that the true value of a school depends upon the method of its instruction. Knowledge must be transferred in a scientific manner if we expect the development of the learner to be normal. There are four recognized modes of imparting knowledge—by lecture, by text-book, by dialogue, by catechization. In the first, the teacher speaks and the pupil listens; in the second, the pupil is mostly in contact with the book; in the third, there is a mutual relation existing, and the fourth leads the pupil from the known to the unknown by a series of questions. Now, the lecture mode is the method generally followed in medical and dental colleges, and a lecture as we usually hear and see it, consists of the entrance of the professor, a nod of recognition, a formal discourse, either oral or written, and then exit of the professor. The student is amazed at the dignity and display of learning, makes a few scattering notes, the hour is gone, and conscious of how little actual knowledge he has gained goes to his room discouraged and discontented. What is the trouble? 1st, The pupil has not come to the school with the preparation that is necessary to make the lecture method a success, because this mode supposes that the class has made itself fully acquainted with the subject as treated in the book, and that the instructor from his experience is able to introduce new thought. 2d, The instructor failed to recognize that the rapid presentation of fact after fact was not consistent with the slow development of the mind; he failed to see the importance of halting to make a single suggestion or illustration.

When a pupil goes to the public school he takes his arithmetic, grammar and geography. The teacher says you may study the examples, rules and problems on page 103; you may study the analysis of the sentences on page 45, and study the physical features of North America. The pupil is not supposed to be acquainted with the thought contained in the lessons, but he is given a chance to do what he can until the time of recitation, when the teachar tests his knowledge, corrects his errors, introduces new facts, discusses principles and makes what other interpretation he deems necessary. Now, since most of the pupils attending a dental college are admitted without any test as to their specific ability, I believe the text-book method, or a judicious combination of the text-book and lecture methods, come as near the ideal as any mode at command. So the student should enter the work with his dental anatomy, dental physiology, dental histology, dental *materia medica*, dental chemistry, his works on irregularities, operative and mechanical dentistry, and that the teacher should say to the class in anatomy, You may study the number and arrangement of the teeth, pages, 1 and 2; To the class in histology, You may study the structure of enamel and dentine; To the class in operative dentistry, You may study the kind of decay and location of cavities, pages 3 and 4. The time of your recitation will be to-morrow from 9 to 10; then I will test and prove your knowledge—supplement the text-book, and do what I can by illustration and explication to enable you to make the subject fully your own. In testing your understanding of the lesson I will ask you a number of questions which shall be clear. They shall be so easy that you will not get discouraged, and so hard that you will have no time for idleness. In proving your understanding of the lesson I shall expect you to answer the questions in an explicit and logical manner. You are not to wander from the point presented in the question, and I want the answer to indicate not a mere committing to memory, but a comprehension—an assimilation of the thought of the text.

Let us consider now, the clinical department of the college. Here, in my judgment, is the most important work of all. In the infirmary the learner is brought in contact with the best of books. Here he sees the text that breathes, lives, feels pain, looks suspiciously or confidently, follows the hand as it selects the instrument, begs for caution, inspects the cleanliness of napkins, finger nails and shirt front, passes judgment, and returns her verdict to every one she meets. Here the man without experience stands in doubt. He approaches the patient for the first time, and realizes for the first time in his life how weak he is. He has studied pictures of chairs in all positions, but he can not determine how high, or at what angle he needs this chair. He moves his nervous hand to the mouth to make a survey; he has seen pictures of decay and cavities, and irregularities, but he has never before seen such cavities and malpositions, so he continues, and at the end of three hours, weary and haggard, he finds to his chagrin that the filling rocks. He is just beginning to appreciate what dentistry means, and to understand what the old dentists say when they enjoin patience and control of temper. We hear many propositions in our daily rounds. Some of them are like these: No man can appreciate a doctrine until he applies it to his own life. You may recognize a person by his external features, but you can not say that you know him unless there has been contact with his inner life or spirit. Now, the pupil has heard theory, doctrine and dogma, but here in this manual work he is applying theory to his own life. He is undertaking something that leaves a more pronounced mark across his soul than any that has hitherto been made. He is engaging in something that tests his nerve, his blood, his sympathy, his judgment and his integrity. In my humble opinion the duties of the infirmary and laboratory are of such gigantic proportions that no boy, or senior student is able, from the standpoint of experience, scholarship, or teacher's qualification to meet the demands that are naturally imposed.

If a person has a friend he wishes to promote, he had better nominate him for any post connected with the college rather than name him for demonstrator, when he is aware that he is a novice and consequently has nothing to exhibit. Colleges talk and print much about their clinical advantages. One reports one thousand clinics during its last session. One has thirty chairs, and another puts the juniors to work immediately, so that no senior has any advantage. Let us see whether a school can treat a thousand patients with equity, or has any scientific plea for putting juniors to work immediately. This department must be founded on the following principles: Like didactics in general, its true value is in method and not in bombast. The patient should have justice; *i. e.*, the best filling, the best crown, the best plate is due her—not the best crown or plate that a junior or senior can make, but the best that any body can make.

The pupil should have justice; *i. e.*, he should derive all the benefit possible from the clinic, and he can not have all possible benefit until the patient has justice. Deduction: The inexperienced pupil alone can not give the patient justice, hence the necessity of the constant attention of the ripest experience. The profession should have justice; this is not possible unless the student and clinic both have justice. The work should be systematic, remembering that the mind develops slowly, and the hand in a corresponding degree. The mind dictates the movements of the hand. A certain amount of mental discipline is necessary before the mind can intelligently dictate, hence the absurdity of allowing beginners to operate until they have received or conceived a design or theory of operation.

The student is not an apprentice—a hewer of wood and drawer of water, but he is a man away seeking knowledge, is paying for his instruction, and is therefore an important factor in the school, and on his needs the requirements of the school rest.

This, then, is my scheme as to this mental-manual training department—this work that brings the hands, the back, the feet, the eyes, the brain, and every part of the pupil into action.

I would have the demonstrator address the class in this manner: Gentlemen, in the other work of the school you have been studying various subjects, and your reason for that is that there is an educational value in such study. You feel that there is a pleasurable value, a practicable value and a disciplinary value. In that work you have been expressing yourselves by oral and written language. You, no doubt, recognize that before you can experience the highest pleasure, possess the greatest degree of practical value, or feel conscious of the strongest mental attainment, that you must express yourselves in a perfected object. You are striving to give a valid illustration of your worth, but you can never do it by writing and talking. You must construct; you must build something. You are probably aware that there are two systems of manual training in this country—one imported from Russia and the other from Sweden. The Swedish system holds that the learner should make useful articles from the first—these articles to have at once some place in the social or domestic life of the pupil.

He would make boot-jacks, saw-bucks, canes, bread-pins and needle-boxes. The Russian system insists that the learner shall not proceed immediately to making articles of utility, but that all work, simple and complicated, shall consist of two steps; First, a drawing; second, construction of the object. The drawing is not a copy of something, but represents principle and processes of construction, and after the completion of the drawing, the construction of the object is accomplished by fulfilling the conditions of the drawing. Now, it is admitted that in the Swedish system there is the advantage of greater immediate success, but the Russian system is of wider range and of larger educational value. We will now proceed with our work, using as much of these systems as the nature of our work will allow. I see you have your clinic registers and pencils. The injunction, "observe, reflect, record," is one that you will now appreciate. Here is our patient; the lady's name is Miss Jay; she resides at 24 Essex street. How many observations do you think we can make in this case? Follow carefully: First, I must calm her fears and gain her confidence. If she is nervous, I am liable to be nervous. If she suffers, I suffer. If I get excited she will be alarmed. Self control is the essential in securing confidence. I must deal gently; I must be steady and deliberate. The second observation will be made before isolating. We find a cavity on the mesial surface of the right upper central; there is no malposition and we can gain sufficient access without separating; the class may come and examine this tooth. We will now indicate in drawing the theory to be followed in preparing the cavity. Please remember that this drawing must set forth conditions to be followed in the use of the instrument. It must show beveled margin, undercut, general or special retention, and degree of excavation. After this is finished, I will call on some one to prepare the cavity according to the principles indicated. I see your drawings are good. Mr. Jones may now come forward and we will get the tooth ready to fill. From this work I would think Mr. Jones appreciates what he placed on paper. Let each member of the class make a careful inspection of this cavity. Notice that there is very little undercut; that the walls are almost at right angles with the bottom of the cavity; every thing is favorable for the use of soft foil.

Before we begin this, we need another drawing—one which sets forth the theory of the filling process. This drawing must show the length of cylinder, its size, its position in the cavity, and means of condensation. After this, some one, with my assistance, will be called to carry out the process represented in the drawing. I have noticed the progress of your work, and feel very much encouraged, but I noticed one that has a wrong idea about the arrangement of the cylinders. He has indicated in his drawing that the small condensed cylinder should be used to start with. Notice the copy which I have on the board; we have indicated the use of a large cylinder, longer than the cavity is deep, to begin with, using smaller and more compact ones as we advance. Mr. Thomas may now come forward and I will help him introduce this filling. Pupils, you have listened attentively to the explanations; you have followed the work in drawing; you have witnessed the preparation and filling of this cavity. It is supposed that you are acquainted with this kind of work; your next duty will be to operate on a similar case. In that work carry out the conditions of your drawing, and heed the instruction which has been given. The class is excused.

The association will note that there is so much to be said and done in conducting a clinic, that all I can do is to mention the principal topics and the order in which they should be discussed. My point is that demonstration is a very difficult work, and therefore requires maturity, scholarship, care, system and constant attention. A teacher's preparation must be determined by the nature of the subjects to be taught and the end to be accomplished by the recipient of the instruction. The person who is professionally able to teach, realizes that true thought in any subject is God's thought, and his task is to make the pupil's thought the same. The professional teacher sees in each of his students a struggle to attain a higher standard. He may see a pupil pursuing something that is not consistent with a perfect ideal, but the inference is that the pupil is mistaken in his idea of manliness, and that his misconception must be corrected by the educational process. The teacher sees an immature judgment, inaccurate observation, uncertain memory, perverted imagination, dishonest intentions and feeble will, and realizes that his mission is to bring order out of chaos.

In this world there are many standards. To excel in physical prowess to conquer nations, to build mansions, to be intellectual, to sway the royal scepter, to extend Christian missions, to comfort the unfortunate, to be like Christ, are some of the prominent standards which control men. What is our standard?

A professional individuality—that purpose, that concentration, that energy that will make us stand in bold relief. That skill that secures and holds confidence; that intelligence which endows with power, wins respect and makes us able to defend; that faith which makes a Christian and that fidelity which makes a patriot. This is the standard of the dental profession, and the embodiment of an ideal college curriculum.

